# From Concept to Clinic: Vepdegestrant (ARV 471) Becomes the First Approved PROTAC Drug

**DOI:** 10.3390/pharmaceutics18070827

**Published:** 2026-07-06

**Authors:** Miklós Bege, Miklós Lovas, Anikó Borbás

**Affiliations:** 1 Department of Pharmaceutical Chemistry, University of Debrecen, Egyetem Tér 1, H-4032 Debrecen, Hungary; bege.miklos@pharm.unideb.hu (M.B.); lovas.miklos@pharm.unideb.hu (M.L.); 2 Doctoral School of Pharmaceutical Sciences, University of Debrecen, Egyetem Tér 1, H-4032 Debrecen, Hungary

**Keywords:** vepdegestrant, PROTAC, breast cancer, Veppanu^TM^, ARV 471, targeted tumor therapy, estrogen receptor, protein degradation, SERD, endocrine therapy

## Abstract

Breast cancer (BC) is a major global public health problem. Classical therapies have limited success on the treatment of BC; therefore, new therapeutic options are needed. Proteolysis targeting chimeras (PROTACs) are heterobifunctional molecules that represent a revolutionary class of new drug candidates because they induce the degradation of harmful, undruggable proteins by activating the ubiquitination machinery of cells. Their unique mechanism of action offers several advantages over conventional drugs, but also disadvantages, as most of them are large molecules with unfavorable pharmacokinetic properties, which limits their bioavailability. Vepdegestrant (Veppanu^TM^) is an orally administered, estrogen receptor (ER) targeting chimera that was approved by the FDA on 1 May 2026, for the treatment of adults with ESR1-mutated advanced or metastatic breast cancer. Thus, vepdegestrant became the first-ever approved PROTAC drug. In this article, we briefly summarize the structure, mechanism of action, and key available pharmacokinetic and pharmacological data of vepdegestrant.

## 1. Introduction

Breast cancer (BC) is the most frequent type of cancer among women and hormone receptor positive (HR+) tumors make up the majority of breast cancer cases [1]. Estrogen receptor alpha (ERα) is a major driver of growth in hormone receptor positive breast cancer, making ER one of the most validated targets in endocrine therapy [2]. Traditional approaches such as aromatase inhibitors (e.g., exemestane, formestane) and selective estrogen receptor modulators (SERMs, e.g., tamoxifen, toremifene) can suppress estrogen signaling, but resistance often emerges through ER mutations, pathway reactivation, or ligand-independent ER activity [3]. This has pushed the field toward approaches that degrade ER rather than merely inhibit it [4].

**Figure 1:**
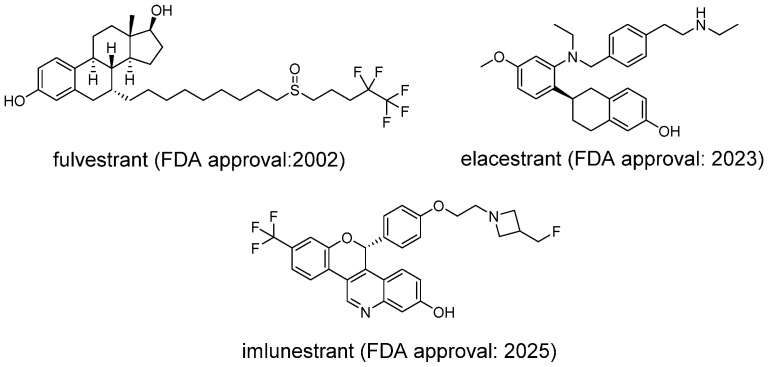
Previously approved SERD medicines.

The first selective estrogen receptor degrader (SERD) was fulvestrant, an estradiol derivative with a large 7*α*-pentafluoroalkylsulfinyl side chain that not only allows it to antagonize the target receptor, but also destabilizes its structure, leading to its degradation by the cell machinery [5] (Figure 1). Although this resulted in a much more drastic reduction in estrogen signaling reduction, its suboptimal pharmacokinetic properties posed problems, requiring intramuscular injections for efficacy [6]. Newer SERDs, such as the recently approved imlunestrant and elacestrant, are designed for oral administration, allowing for more convenient dosing, and are also effective against ESR1-mutated, endocrine-resistant breast cancer [7].

Fulvestrant, and SERDs in general, represent an early iteration of an emerging approach to drug design: targeted protein degradation (TPD), which shifts the focus from designing inhibitors to designing molecules capable of hijacking the various proteolytic mechanisms required for the degradation of selected proteins [8]. Many different modalities are known by now, each with its own scope, advantages and disadvantages, but by far the most prominent is the PROTAC (proteolysis targeting chimera) modality [9].

**Figure 2:**
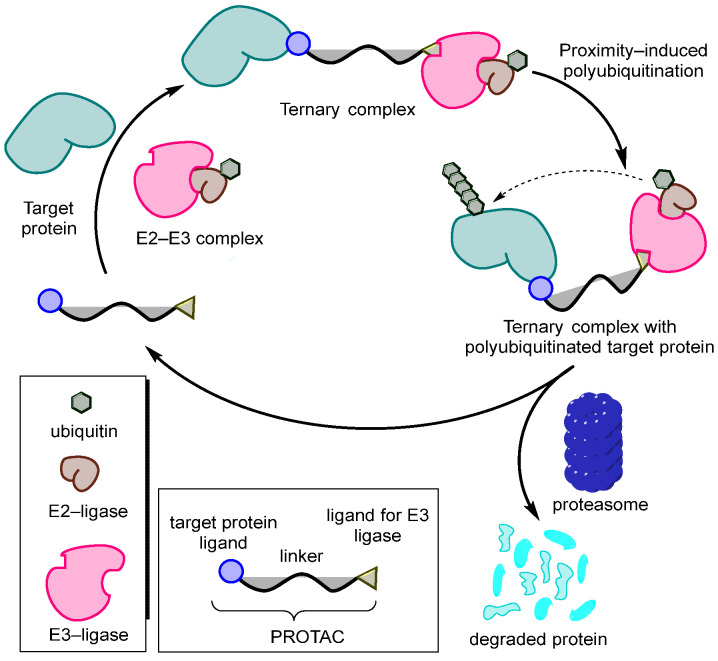
Mechanism of action of PROTACs.

PROTACs are heterobifunctional molecules consisting of an E3-ligase ligand and a target protein ligand connected by a linker (Figure 2). E3 ligases constitute a large family of enzymes responsible for the ubiquitination of proteins, which can result in changes in protein localization, activity, interactions, or, most importantly, degradation by the 26S proteasome. PROTACs hijack this system by simultaneously binding to both the target protein and a selected E3 ligase, creating a ternary complex; this forced proximity enables the polyubiquitination and subsequent degradation of the target protein [9]. The PROTAC molecule can then participate in the formation of further ternary complexes, acting catalytically, in contrast to traditional inhibitors, which generally act stoichiometrically, with each molecule inhibiting a single protein [10]. The main features of PROTAC-, SERD-, and SERM-based ER-targeting approaches are summarized in Table 1.

Besides the catalytic mechanism and the complete knock-out of the target protein, PROTACs offer other advantages: they can be used against targets previously described as “undruggable” (proteins without good binding pockets), they can overcome different resistances and they can offer higher target selectivity over conventional inhibitors by ternary complex optimization [11].

**Figure 3:**
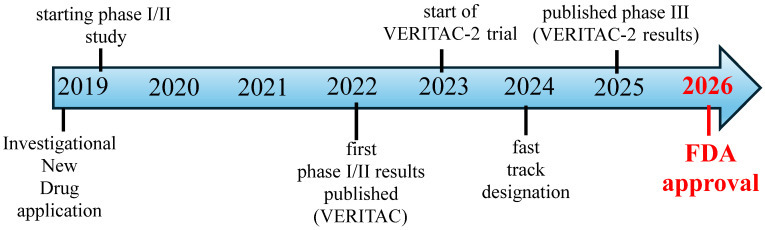
Milestones in the development of vepdegestrant.

Dozens of PROTACs with different targets are currently in clinical trials [12], and fittingly, the first to recently gain FDA-approval was a new SERD designed to overcome resistance mechanisms that limited earlier endocrine therapies, vepdegestrant (Figure 3).

PROTACs are not only specific in their mechanism of action but also in their resistance mechanism. Unlike traditional inhibitors, where resistance generally arises from downregulation/mutation of the target protein, PROTAC-resistance may instead arise from changes in the ubiquitin–proteasome system (UPS), specifically the downregulation/loss-of-function mutations of key components such as targeted E3 or E2 ligases or the members of the COP9 signalosome complex [13]. In the case of vepdegestrant, loss-of-function mutations or downregulation of its targeted E3 ligase, CRBN, may be an important factor in drug resistance. Similar to how CRBN expression levels correlate with better survival outcomes in multiple myeloma patients treated with IMiDs, the efficacy of vepdegestrant may also correlate with CRBN levels and therefore may be worth monitoring [14,15].

Another possible resistance mechanism, which is shared with conventional inhibitors, is efflux by pumps such as MDR1 [16]. Consequently, it is hypothesized that concomitant blockade of MDR1 may enhance the efficacy of PROTACs.

## 2. Vepdegestrant

### 2.1. Chemistry and Drug-Likeness

**Figure 4:**
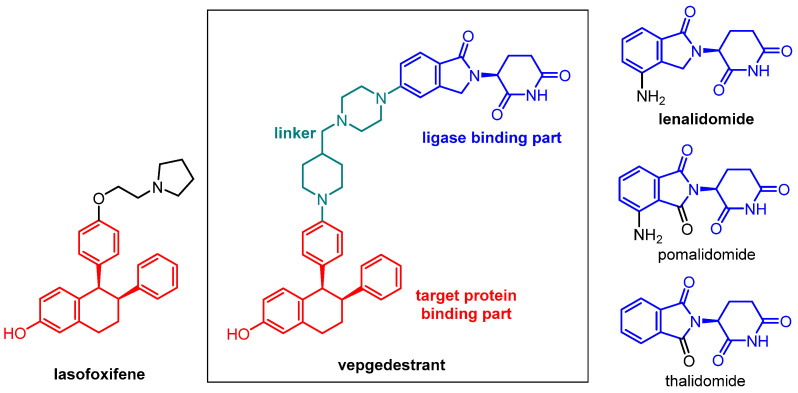
Structural elements of vepdegestrant.

Like other PROTACs, vepdegestrant consists of three main parts: a target protein (ER) binding moiety, a linker and a ligase binding moiety (Figure 4). The first unit, responsible for binding to the ER is structurally similar to the 3rd generation SERM, lasofoxifene, which contains a 6-hydroxi-tetrahydronaphthalene skeleton. The linker consists of two heterocycles, a piperidine and a piperazine, making the structure more rigid, which increases efficacy by allowing for more stable ternary complex formation: their constrained geometry minimizes conformational entropy, placing the target protein and the E3 ligase in an optimal orientation for efficient ubiquitin transfer. Additional advantages of this linker include improved pharmacokinetic properties and greater metabolic stability compared to alkyl- or ethylene glycol-based linkers. The cereblon E3 ligase (CRBN) binding moiety of vepdegestrant is a glutarimide isoindolinone derivative that shares similarities with thalidomide and lenalidomide [17,18].

In terms of drug-likeness properties, vepdegestrant violates the Lipinski rule of five in two respects, as its lipophilicity is above 5 and its molecular weight is between 500 and 1000 (Table 2). However, it is not unprecedented that an orally available drug is found in this molecular weight range. Vepdegestrant has advantages in other respects. Despite its size, vepdegestrant contains only a small number of hydrogen bond donors (HBDs) and hydrogen bond acceptors (HBAs), and its polar surface area is below 140 Å^2^. Due to the rigid linker, the number of rotatable bonds is also small. All of these may contribute to the good permeability and oral bioavailability of vepdegestrant [17].

Various approaches exist to overcome the general pharmacokinetic difficulties of PROTACs, including chemical (structure optimization, e.g., using rigid linkers) and formulation methods. The formulations mostly consist of some kind of nanoformulations including polymer, lipid, and gold nanoparticles, or nanoliposomes, etc. [19]. Little information is available on the formulation development of vepdegestrant. Niessen et al. reported that formulations generating amorphous nanoparticles improved absorption through particle drift effects. In this way, permeability increased up to seven-fold compared to non-colloidal amorphous powder [20].

### 2.2. Pharmacodynamics and Preclinical Results

Vepdegestrant (PF-07850327 or ARV 471) simultaneously binds to ERα and CRBN, forming a ternary complex. As a result of the complex formation, CRBN ubiquitinates ERα, leading to its degradation by the 26S proteasome. In the absence of ERα, the estrogen-dependent signaling cannot function properly, thereby blocking the growth of estrogen-dependent tumors. After protein degradation, vepdegestrant is released and can bind to another target molecule. A major advantage of this mechanism of action is that it is effective against mutations such as Y537S or D538G, which confer resistance to conventional anti-ER strategies (e.g., Y537S or D538G) [17]. The mechanism of action was examined and demonstrated in multiple levels. The formation of the ternary complex was confirmed by CRBN:probe displacement assay. The ternary complex CRBN-DDB1:ER-LBD:vepdegestrant was isolated, and the structure was studied by single-particle cryo-EM. The dominance of ER degradation over ER inhibition was investigated using A5927 (an E3 inactive ER-degrading PROTAC), which showed a ten-fold lower activity (IC_50_ = 33.23 nM/L vs. 3.057 nM/L) compared to vepdegestrant (luciferase target engagement assay). Addition of other CRBN-binding (lenalidomide), or ER-binding (lasofoxifene) molecules in 10-fold excess inhibited ER degradation induced by vepdegestrant, as did the addition of proteasome inhibitor (carfilzomib), supporting the proteasome-induced degradation mechanism [14]. In cell-free assay, vepdegestrant bound to recombinant ER with an IC_50_ of 0.99 nmol/L and a K_i_ of 0.28 nmol/L. These results are comparable to those of diethylstilbestrol and lasofoxifene [21].

In T47D-KBluc cellular luciferase assay, vepdegestrant dose-dependently decreased estradiol-bound ER/ERE-driven luciferase expression after 24 h of treatment with an IC_50_ value of 1.1 nmol. Vepdegestrant dose-dependently induced loss of wt ERα in MC7F cells (DC_50_ = 0.9 nM, D_max_ = 95%), achieving above 80% reduction in ERα at 100 nM within 4 h. Western blotting revealed that ER level was also decreased in BT474, CAMA-1, ZR-75-1, and T47D cell lines (patient derived BC cells). Vepdegestrant reduced the levels of clinically relevant mutant ERs (Y537S, D538G, Y537C, Y537N, E380Q, L536P, V422del) in T47D cells [21].

Vepdegestrant showed remarkable selectivity toward ER. Analysis of the MCF7 proteome after vepdegestrant treatment (7 h, 10 nmol/L) revealed a significant decrease in ER levels, but also a small decrease in PGR (progesterone receptor) and growth regulating estrogen receptor binding 1 (GREB1) levels. The observed mild vepdegestrant-mediated degradation of PGR (IC_50_ of 17 nmol/L compared to 1 nmol/L IC_50_ value against ER) may be due in part to transcriptional downregulation of the ER-responsive genes, as both PGR and GREB1 RNA levels were reduced [21].

The GI_50_ values were 3.3 nM and 4.5 nM in MCF7 and T47D cells, respectively, with wild-type ER after 5 days. T47D cells expressing Y537S and D538G were slightly less sensitive, with GI_50_ values of 8 and 5.7 nmol/L, respectively [17,21].

Vepdegestrant at 10 mg/kg three times daily reduced tumor ER levels by at least 90% in MCF7-bearing mice. Vepdegestrant at 30 mg/kg did not show any inherent ER agonist effect but decreased ER levels in uterine tissue by 76%, while fulvestrant showed a 65% decrease in the same assay. Vepdegestrant showed robust tumor growth inhibition (TGI) in MCF7 tumor xenograft mice models (85%, 98% and 120% TGI at 3, 10 and 30 mg/kg daily dose respectively). Above 94% ER levels, a decrease in tumor cell lysate was observed. Vepdegestrant 10 and 30 mg/kg daily and fulvestrant 200 mg/kg (twice weekly for 2 weeks and once weekly for 2 weeks) were administered for 28 days in a Y537S mutant xenograft model. TGI was 99% and 107% for the two doses of vepdegestrant and 62% for fulvestrant. ER reduction was 88% for 30 mg/kg of vepdegestrant and 63% for fulvestrant, respectively. Vepdegestrant was also active against palbociclib- and abemaciclib-resistant tumors and showed synergy with CDK4/6, mTOR and PI3K inhibitors [21].

### 2.3. Pharmacokinetics, Stability and Interactions

Due to their dual targeting structure, PROTACs are inherently large, complex molecules with high molecular weight, many rotatable bonds and potential cleavage sites during metabolism. PROTACs generally do not comply with Lipinski’s rule of five and Veber’s rule; therefore, they are expected to have poor oral bioavailability [15]. Due to the aforementioned issues, pharmacokinetics (PK) is a crucial aspect of PROTAC-based drug candidates; therefore, PK properties of vepdegestrant have been extensively studied (see Table 2) [20,22,23,24].

A sensitive LC-MS/MS method was developed and validated by Niessen et al. for the quantification of vepdegestrant in rat plasma for stability and PK studies. Vepdegestrant was shown to be stable in rat plasma after 20 min of storage at room temperature in the dark (slight instability was detected when exposed directly to light), and after 7 weeks of storage at −20 °C. A methanolic solution of vepdegestrant was also stable at −20 °C for 8 weeks. After longer storage at room temperature, cleavage of the glutarimide ring is likely to occur. After a single i.v. dose of 1 mg/kg of vepdegestrant, the AUC_0–6h_ was 585.4 ± 43.82 h·ng/mL, while after intraduodenal bolus administration, only 73.3 ± 3.8 h·ng/mL AUC_0–6h_ was achieved, indicating low bioavailability (F = 12.5 ± 0.6%) [23].

In another publication, the Niessen group demonstrated that vepdegestrant undergoes enzymatic degradation in diluted rat intestinal fluid in vitro, in which serine proteases probably play an important role, as adding a serine protease inhibitor doubled the half-life (from 5.9 ± 3.8 h to 13.9 ± 4.5 h). In rat serum, the t_1/2_ of vepdegestrant was measured to be 3.5 ± 1.5 h. The effective intestinal permeability (P_eff_) of vepdegestrant was measured in the presence of a serine protease inhibitor. Without ketoconazole, P_eff_ was 0.23 ± 0.08 × 10^−4^ cm/s, while the addition of ketoconazole increased it more than two-fold (to 0.53 ± 0.15 × 10^−4^ cm/s). Since ketoconazole is a P-gp inhibitor, the results suggest a major role of P-gp-mediated efflux in the low intestinal permeability of vepdegestrant. After a single dose (1 mg/kg) intraduodenal (id) administration, the C_max_ of vepdegestrant was 18.0 ± 8.1 ng/mL, the t_max_ was 1 h, and the AUC_0–6h_ was 74.4 ± 12.2 h·ng/mL. Administration of the P-gp inhibitor ketoconazole (vepdegestrant C_max_ = 25.9 ± 13.1 ng/mL, t_max_ = 1.5 h, AUC_0–6h_ =124.4 ± 30.9 h·ng/mL) or encequidar (C_max_ = 23.4 ± 8.0 ng/mL, t_max_ = 2.0 h, AUC_0–6h_ = 109.0 ± 15.3 h·ng/mL) increased these values; however, the increase in relative bioavailability was nonsignificant. Ketoconazole did not change the plasma AUC of vepdegestrant after i.v. administration. The concentration of vepdegestrant was higher in bile than in plasma, indicating that it is either eliminated with bile or undergoes enterohepatic circulation. The major elimination pathway is degradation by plasma hydrolases, while CYP3A plays a minor role in elimination [22].

A Korean group developed an HPLC-MS/MS method for the quantification of vepdegestrant in biological samples [24]. The stability of vepdegestrant showed pH-dependence, as it remained at 82.6% in acidic (pH = 2 puffer) solution at 37 °C for 2 h, while at pH 4–10, the amount of vepdegestrant decreased to 2.25–12.8% within 2 h. Considering that the adsorption of vepdegestrant to the container surface is also pH-dependent, the decreased amount of vepdegestrant between pH 2–8 can be explained, at least in part, by adsorption. In mouse and rat plasma, vepdegestrant was stable for 4 h at 4–37 °C. This higher stability possibly can be explained by plasma protein binding, which also prevents adsorption to the container surface. Differences in the adsorption to the test tube wall were investigated, and intestinal absorption may be enhanced at low lumen pH due to enhanced apparent solubility. Vepdegestrant showed high stability in dog, mouse and human microsomes, but low to moderate stability in rat microsomes (32.2% remained after 60 mint incubation). After i.v. administration of 2 mg/kg, clearance (CL) was 313.3 ± 44.2 mL/h/kg, the AUC_inf_ was 6.507 ± 1.057 µg/h/mL in mice. In rats, the CL was 1053 ± 49 mL/h/kg and AUC_inf_ was 1.902 ± 0.090 µg/h/mL, and the t_1/2_ was 3.970 ± 0.284 h. After 5 mg/kg oral dose, the AUC_inf_ was 2.913 ± 0.707 µg/h/mL, the t_1/2_ was 3.637 ± 1.399 h in mice, and these values were 1.147 ± 0.446 µg/h/mL and 4.068 ± 0.418 h in rats respectively. The F value (bioavailability %) was slightly higher in rats than in mice (24.12 ± 9.39% vs. 17.91 ± 4.35%). The CL was lower in mice than in rats, which was expected based on the metabolic stability tests mentioned above. The possibility of an intestinal first pass effect has also been raised [20,24].

Vepdegestrant shows high protein binding in human and rat plasma, hepatocytes and liver microsomes. After incubation with hepatocytes, hepatic extraction ratios were 69.22% and 41.47% in humans and rats, respectively, while they were 27.62% and 48.63% after incubation with liver microsomes. These data indicate low to moderate hepatic clearance, which is consistent with other results suggesting that the major metabolic pathway is protease-mediated degradation. After oral administration of 20 mg/kg ^14^C-labeled vepdegestrant, the t_max_ of total radioactivity (TRA) was 2.67 h, the C_max_ was 2.197 ng eq./mL, the AUC_0–∞_ was 25.036 ng eq./(mL/h), and the t_1/2_ was 12.2 h. These data are not comparable to previous results due to the different conditions (e.g., dose). There were also differences between the PK of the parent drug and the radiolabeled derivative. For example, vepdegestrant showed higher AUC_0–∞_ and t_1/2_ in female rats than in males, while no difference of these values was observed in TRA. More than 90% of radioactive substances excreted through feces, with <2% urine excretion. The radioactive substances were distributed widely among tissues. After 48 h, the highest radioactivity was measured in the eye, indicating potential safety issues. Faster hepatic metabolism was observed in female than male rats. In fed rats, absorption of vepdegestrant was slower than in fasted ones, but the absorbed amount was higher. Eleven metabolites were identified, the main metabolites being oxidized, hydrolyzed and glucuronidated derivatives. Cleavage of the linker has also been observed [25].

In another study, electron-activated dissociation HRMS was used to identify vepdegestrant metabolites. After incubation with canine microsomes, 12 phase I metabolites and glucuronides were detected. The main identified biotransformations were: piperazine-*N*-dealkylation, hydrolysis of the glutarimide ring, phenol-*O*-glucuronidation, piperidine oxidation and piperidine lactam formation [26].

In the oxidative metabolism of vepdegestrant, CYP3A4 plays a major role. Therefore, in the NCT06005688 phase I study, the effect of CYP3A4 inducer (carbamazepine) on vepdegestrant PK was investigated in 12 healthy adult male participants. Administration of multiple doses of carbamazepine after a single dose of vepdegestrant decreased the AUC_inf_ of vepdegestrant to 64.1% and the C_max_ to 80.2% compared to vepdegestrant alone. The T_max_ was 6.0 h in both cases, and the t_1/2_ was decreased from 50.1 h to 45.4 h with carbamazepine. These results indicate that CYP3A4 is involved, but does not play a major role in the elimination of vepdegestrant [27].

In the NCT05538312 phase I study, the effect of the CYP3A4 inhibitor itraconazole on the PK of vepdegestrant was investigated in 12 participants. AUC_inf_ was increased by 68.9%, while C_max_ was increased by 52.2%. Also, t_1/2_ was increased from 42.6 h to 61.6 h, but this may be a consequence of unequal sampling. These changes are significant, albeit much smaller than those observed for drugs primarily eliminated by CYP3A4, supporting previous findings that although vepdegestrant is a substrate of CYP3A4, it is not the primary route of elimination. These changes in PK are unlikely to affect the safety of vepdegestrant [28]. It is possible that these changes are due to the P-gp inhibitory effect of itraconazole, as Niessen et al. have reported similar PK changes, using P-gp inhibitors [22,29].

Based on in vitro results, it was assumed that vepdegestrant is an inhibitor of P-gp and breast cancer resistance protein, BCRP. The effect of vepdegestrant on the PK of dabigatran, a P-gp substrate, was studied in a phase I study in 24 healthy participants (NCT05673889). Vepdegestrant increased the AUC_inf_ of dabigatran etexilate by 97.8% and the C_max_ by 92.2%. The effect of vepdegestrant on the PK of rosuvastatin, a BCRP substrate, was also studied in on a total of 12 participants (NCT05652660). The AUC_inf_ and C_max_ of rosuvastatin was also increased moderately (16.9% and 20.5% respectively [30].

The interaction between vepdegestrant and esomeprazole (NCT06275841) and midazolam (NCT06256510) was also studied [17].

In Japanese patients, the median T_max_ was 4.74 after a single dose and 4.69 h after multiple QD (quaque die = once daily) doses (200 mg daily). C_max_ was 630.9 ng/mL after a single dose and 1056 ng/mL after multiple doses. AUC_24_ was 10.40 ng∙h/mL after single dose and 18.31 ng∙h/mL after multiple doses. The accumulation ratio R_ac_ was 1.76 and the effective elimination half-life (t_1/2eff_) was 20.2 h [31]. Based on the above, as high-fat meal increased AUC (2.9-fold) and C_max_ (3.2-fold), it is recommended that vepdegestrant be taken with food.

No specific food–drug interaction was identified; however, based on theoretical considerations, as vepdegestrant is a CYP3A substrate, consumption of CYP3A inhibitors (e.g., certain fruits) should be avoided. For example, CDK4/6 inhibitors (which are an important part of modern breast cancer treatment protocols) are metabolized by CYP3A, palbociclib, abemaciclib, and ribociclib are substrates of P-gp, and palbociclib and abemaciclib are substrates of BCRP [32,33]. The fact that vepdegestrant is a CYP3A substrate and acts as an inhibitor for P-gp and BCRP raises questions, as the therapy of advanced/metastatic BC consists of multiple drugs, increasing the risks of potential interactions. For example, CDK4/6 inhibitors, which are important parts of modern BC treatment protocols, are metabolized by CYP3A, and palbociclib, abemaciclib and ribociclib are substrates of P-gp, and palbociclib and abemaciclib are also substrates of BCRP [32,33].

It is also important to consider that the main target population of vepdegestrant is elderly patients, who are generally more susceptible to drug–drug interactions (DDIs) as they often take multiple medications for their comorbidities, such as antihyperlipidemic agents (e.g., rosuvastatin), anticoagulants (e.g., dabigatran), antihypertensives (e.g., verapamil), etc. The known results have not revealed any safety concerns so far when vepdegestrant is co-administered with P-gp and BCRP substrates such as dabigatran and rosuvastatin. On the other hand, the pharmacokinetics of these drugs upon co-administration were moderately to greatly altered, which is of some concern, especially since the published studies used a single dose of vepdegestrant, leaving the safety implications of long-term co-administration unclear [17,30]. Further studies are definitely needed in this regard, especially in the longer term. In general, concomitant use of vepdegestrant with CYP3A inhibitors or inducers should be avoided. If unavoidable, dose reduction may be necessary in the former case and dose increase in the latter case to maintain vepdegestrant levels within the therapeutic window. It is also recommended to avoid concomitant use of vepdegestrant with P-gp substrates, as even minimal dose increases may cause severe toxicity due to vepdegestrant increasing exposure to P-gp substrates by inhibiting P-gp.

### 2.4. Efficacy in Clinical Trials

In a phase I dose-escalation trial (NCT04072952), 83 previously treated patients with ER+HER2− advanced breast cancer received 30–700 mg of vepdegestrant daily. A reduction in mutant ESR1 circulating tumor DNA was observed. The clinical benefit rate was 37% after 24 weeks [34]. In the phase II cohort extension of the study (called VERITAC) 2 doses (200 mg and 500 mg QD) were tested. The clinical benefit rate (CBR) was 37.1% (n = 35), and the objective response rate was 8.3% (n = 24). The median progression-free survival was 3.5 months in all evaluable patients. Because of comparable efficacy, the 200 mg daily dose was chosen for further evaluation [35,36,37]. In the phase Ib cohort of the study, the combination of vepdegestrant–palbociclib (a CDK4/6 inhibitor) was investigated in 46 patients. Doses of 180–500 mg of vepdegestrant and 125 mg of palbociclib were administered QD for 21 days followed by 7 days of treatment-free interval, setting up a 28-day cycle. The CBR was 63%, indicating a clinical benefit of this combination as expected based on preclinical results. The objective response rate (n = 31) was 41.9% [38].

In the NCT05463952 phase I trial, six female Japanese patients with ER+ HER2− advanced breast cancer received 200 mg once daily dose of vepdegestrant for 6–28 weeks. Two patients had stable disease at week 24 and four patients had disease progression. Limitations of the study are the small sample size, lack of control, short follow-up period, and the heavily pretreated patients [31].

VERITAC-2 (NCT05654623) is a global, multicenter, randomized (randomization was stratified according to ESR1 mutation status and presence or absence of visceral disease), unblinded, open label phase III trial, comparing the efficacy and safety of vepdegestrant and a well-known SERD, fulvestrant, in ER+, HER2− advanced BC, after CDK4/6 inhibitor + endocrine therapy. Median progression-free survival as a primary endpoint was assessed by blinded independent central review for patients with ESR1 mutations and total patient groups. A dose of 200 mg of vepdegestrant was per os applied QD in each 28-day cycle to 313 patients (136 with ESR1 mutations). Fulvestrant 500 mg was administered intramuscularly on the 1st and 15th days of cycle 1 and on the 1st day of each following cycles to 311 patients (134 with ESR1 mutations). Median progression-free survival was significantly longer in the vepdegestrant group than in the fulvestrant group (5.0 months with 95% CI: 3.7–7.4 vs. 2.1 months with 95% CI: 1.9–3.5) among patients with ESR1 mutations (hazard ratio, 0.58 [95% CI, 0.43–0.78]; *p* < 0.001), but not in the full patient population, were progression-free survival was 3.8 months (95% CI: 3.7–5.3) vs. 3.6 months (95% CI: 2.6–4.0 (hazard ratio, 0.83 [95% CI, 0.69–1.01]; *p* = 0.07)). The objective response rate was 18.6% (95% CI, 12.1–27.4) vs. 4.0% (95% CI, 1.6–9.8) among ESR1 mutant patients. A limitation of the study is the relatively short follow-up period [39]. Trovato and Tortora noted the lack of results in the ER wt subgroup and performed an analysis based on the results, which showed that the median progression-free survival was 2.7 months in the vepdegestrant group compared to 4.8 months in the fulvestrant group in patients with wt ER, suggesting that fulvestrant was superior in this subgroup and highlighting differences between wild-type and ER mutant tumors. However, the results of this analysis should be treated with caution. Shimoi and Yonemori also highlighted the importance of the type of ESR1 mutation, which can have serious consequences for the efficacy of the drugs tested. Further analysis of the mutations included in the study would be useful [40].

### 2.5. Comparison of the Efficacy of Vepdegestrant and Oral SERD Drugs

Elacestrant and imlunestrant are the first two oral SERDs approved for the treatment of specific types of advanced or metastatic breast cancer. Elacestrant (Orserdu) was approved by the FDA in 2023 for the treatment of ER+, HER2−, ESR1-mutant advanced/metastatic breast cancer in postmenopausal women or adult men. The recommended dose is 345 mg once daily. Patients with progressive disease who had received prior endocrine therapy, including the CDK4/6 inhibitor fulvestrant or an aromatase inhibitor, were enrolled in the phase III study with a median follow-up of 15.1 months. Progression-free survival was 2.8 months in the elacestrant group in the overall population and 3.8 months in the ESR1 mutant subgroup, compared with 1.9 months in the control group, who received standard care, which included either fulvestrant or an aromatase inhibitor. The hazard ratio between elacestrant and fulvestrant was 0.68 (95% CI: 0.52–0.90 *p* = 0.0049) in all patients and 0.50 (95% CI: 0.34–0.74 *p* = 0.0005) in the ESR1 mutant group [41,42].

Imlunestrant (Inluriyo) was approved by the FDA for the treatment of adults with ER+, HER2−, ESR1-mutated advanced/metastatic breast cancer whose disease has progressed after at least one line of endocrine therapy. Patients were studied whose disease had progressed after first-line therapy and an aromatase inhibitor (with or without CDK4/6) in the adjuvant or neoadjuvant setting. Patients received imlunestrant monotherapy, standard endocrine therapy (i.e., exemestane or fulvestrant), or imlunestrant in combination with abemaciclib. Progression-free survival was 5.6 (95% CI: 5.3–7.3) months versus 5.5 (95% CI: 4.5–5.6) months (imlunestrant vs. standard), while in the ESR1 mutant subgroup it was 5.5 (95% CI: 3.9–7.4) versus 3.8 (95% CI: 3.7–5.5) months. The abemaciclib-imlunestrant combination was superior to imlunestrant monotherapy [43,44].

In the phase III study, progression-free survival was significantly longer in patients treated with vepdegestrant than in patients treated with fulvestrant in the ESR1 mutant subgroup, but not in the overall population or the ER wild-type subgroup. In the pre-approval clinical trials, fulvestrant was used as a control, which is an older drug with less favorable pharmacokinetics and reduced efficacy against ESR1 mutation. More modern SERDs, such as elacestrant and imlunestrant, have shown similar results to vepdegestrant in clinical trials. However, caution should be exercised when directly comparing these results, as although the main conditions (e.g., sample size, primary endpoint, patient population) were similar in these studies, there were several important differences (e.g., shorter follow-up period in the VERITAC-2 study or differences in exclusion criteria). Some important parameters of the studies, as well as the efficacy of the primary endpoints, are summarized in Table 3. To accurately assess the advantages and disadvantages of vepdegestrant versus SERDs, a clinical trial that directly compares vepdegestrant with a modern, oral SERD is desirable.

### 2.6. Adverse Effects

In a dose-escalation study (NCT04072952), treatment-related adverse effects (TRAE) were observed in 70 of 83 patients. The most common TRAEs (observed in more than one patient) were nausea, fatigue, headache, arthralgia, constipation and hot flush. Most adverse effect was grade 1 or 2, with no grade 4 or higher adverse effects observed. Overall, vepdegestrant was well tolerated and there was no dose-limiting toxicity [25]. In the phase II expansion of the study, the most common TRAEs were fatigue, hot flush, nausea, arthralgia, increased aspartate aminotransferase and increased blood alkaline phosphatase (grade 1 or 2). No dose reduction was needed; however, two patients (5.7%) had to discontinue the treatment due to grade 3 QT elongation and grade 3 anemia [35,36,37]. In the phase Ib cohort, the effect of vepdegestrant + palbociclib was studied. No dose-limiting toxicity was observed. Among the 46 patients, TEAEs (treatment-emergent adverse events) lead to dose reduction of vepdegestrant in five cases and to discontinuation of vepdegestrant in four cases. TEAEs leading to dose reduction or discontinuation of palbociclib occurred in 34 and eight patients, respectively. The most common (>10%) grade 3/4 TRAEs were neutropenia (89.1%), decreased white blood cell count (15.2%), and decreased platelet count (10.9%). No grade 5 TRAEs was observed, and no patient had febrile neutropenia. Overall, the safety profile was similar to the previous data, except for grade 3/4 neutropenia, which required monitoring and dose reduction of palbociclib [38].

In the NCT05463952 phase I trial, no serious adverse effects (AE) were reported; four out of six patients experienced grade 1 or grade 2 treatment-emergent adverse effects. No dose-limiting toxicities was observed [31].

In the NCT0600568 phase I trial in 12 healthy male participants, 50% of patients reported a total of nine TEAEs after a single dose of vepdegestrant (200 mg), and no severe adverse effects were observed. About 75% of patients reported a total of 20 TEAEs after a single dose of vepdegestrant and multiple doses of carbamazepin. In 16.7% of participants, increased hepatic enzyme levels were reported, probably due to carbamazepine [27].

In the NCT05673889 trial, only mild or moderate TEAEs (e.g., headache) were reported. In the NCT05652660 trial, mild dizziness and headache were reported. There were no clinically meaningful changes in vital signs, ECGs or laboratory measurements [30]. In the NCT05538312 trial, one participant suffered mild abdominal pain and diarrhea after a single dose of vepdegestrant [28]. In the VERITAC-2 trial, 86.9% of patients in the vepdegestrant group and 81.4% in the fulvestrant group reported adverse effects. In the vepdegestrant group, the most common AEs were fatigue (26.6%), increased aspartate and alanine aminotransferase level (both 14.4%) and nausea (13.5%). Grade 3 adverse events occurred in 19.2% of patients in the vepdegestrant group and 14% in the fulvestrant group, while grade 4 AEs occurred in 1.6% and 2.9% of cases, respectively. The most frequent grade 3/4 AEs were neutropenia (1.9%) and hypokalemia (1.9%) in the vepdegestrant group. In 9.9% of vepdegestrant patients, QT prolongation was reported, leading to dose reduction in one patient. Overall, in the vepdegestrant group, 1.9% of cases led to dose reduction and 2.9% to treatment discontinuation [39].

Based on the above, the main safety concerns of vepdegestrant treatment are neutropenia and the risk of QT prolongation. Neutropenia is a common, serious adverse effect of many antitumor agents, which can have a major impact on patient well-being and increase the risk of infections and febrile neutropenia. The neutropenia caused by vepdegestrant is possibly related to the lenalidomide analogue part of the molecule, as immunomodulatory thalidomide derivatives are known to cause neutropenia, probably by downregulating the transcription factor PU.1, which plays a key role in granulocyte differentiation, among other mechanisms [45,46]. Interestingly, grade 3 or higher neutropenia was reported with imlunestrant treatment (2.1% vs. 1.9%) and in the fulvestrant group of the VERITAC-2 trial (1.0%), whereas no neutropenia was reported with elacestrant treatment. This suggests that neutropenia is a possible but not a necessary side effect of ER degrader therapy [39,41,42,43,44,47]. It is also noteworthy that the risk of neutropenia is generally higher when ER degraders are used in combination with CDK4/6 inhibitors (e.g., vepdegestrant + palbociclib, or imlunestrant + abemaciclib). CDK4/6 inhibitors, in combination with endocrine therapy, are important agents in the treatment of HR+, HER2− BC and are well known for their risk of neutropenia. In view of this, the combination of vepdegestrant and other ER degrades with CDK4/6 inhibitors requires special caution and, if necessary, dose reduction [48,49,50]. General management of neutropenia associated with cancer treatment includes careful monitoring, dose reduction, antibiotic prophylaxis, or the use of G-CSF (recombinant granulocyte colony-stimulating factor) [45,46].

QT prolongation is another significant adverse event, with dose reductions and treatment discontinuation reported, although most QT prolongation was mild (mean increase from baseline 11.1 ms, upper 90% confidence interval 13.7 ms). QT prolongation was much more common with vepdegestrant group than with fulvestrant (overall 9.9% vs. 1.3%, grade 3 1.6% vs. 0.3%) [39]. Among other SERDs, no cardiac toxicity was observed with elacestrant; however, patients with cardiac disease, including QT prolongation, were excluded from the EMERALD trial, which may have affected this result [42]. In case of imlunestrant, bradycardia was reported in 2.1% of patients. In the SERENA-6 trial of the investigational SERD camizestrant, 5.2% of patients experienced bradycardia [44]. Another investigational SERD, giredestrant also showed cardiac adverse effects; however, less data are available. In a zebrafish model, giredestrant and camizestrant induced bradycardia by modulating nuclear ERα signaling. Interestingly, the esr1 mutation (the zebrafish analog of ESR1) provided protection against SERD-induced bradycardia, but this does not mean that the ESR1 mutation reduces the cardiotoxicity of ER degraders, as these mutations usually only affect tumor tissues and not the heart [51,52,53].

Based on the above, neutropenia is likely an off-target effect, and vepdegestrant has a similar risk to imlunestrant but a higher risk than fulvestrant. However, clinically approved and widely used anticancer drugs, CDK4/6 inhibitors, have a much higher risk of neutropenia, and there are well-established methods for treating neutropenia in cancer patients. The cardiotoxicity of vepdegestrant, on the other hand, can be based on an on-target effect of Erα signaling inhibition. Due to the lack of sufficient literature data, comparison with more modern SERDs is difficult, but QT prolongation is more frequent with vepdegestrant, than with fulvestrant. However, fulvestrant is known as a SERD with lower cardiac effect, so a direct comparison of the effect of imlunestrant or elacestrant would be useful. Concomitant use of vepdegestrant with other drugs known to prolong QT should be avoided, and dose reduction may be necessary if the QTc change exceeds 60 ms, and discontinuation of treatment if the QTc change exceeds 500 ms. Overall, these adverse reactions warrant caution, but do not question the use of vepdegestrant.

Other common adverse events are summarized in Table 4. It can be seen that there is a high overlap between the adverse effects of vepdegestrant and other approved ER-degrading agents, and in most cases the frequency of these events is similar for vepdegestrant and the SERDs. It is important to note, however, the limitations of this literature comparison. In short, vepdegestrant is considered to have a favorable safety profile.

## 3. Discussion of Current Status and Future Perspectives

Vepdegestrant received FDA fast-track designation in 2024 [24]. In 2025, Arvinas and Pfizer Inc. have submitted New Drug Application (NDA) for vepdegestrant to FDA, for the treatment of patients with ESR1 mutant ER+/HER2− advanced or metastatic BC whose disease progressed after endocrine therapy [54]. On 1 May 2026, the FDA approved vepdegestrant for the treatment of adults with ER+, HER2− ESR1 mutated advanced or metastatic breast cancer, as detected by an FDA-authorized test, with disease progression following at least one line of endocrine therapy [55].

The approval of vepdegestrant is significant not only because it was the first clinically approved PROTAC drug, but also because of the regulatory framework used to evaluate it. While the results were mixed in terms of effectiveness in the overall study population, efficacy in a biomarker-defined subgroup (ESR-1 mutation) was significant, and the FDA’s approval based on this represents a move toward precision regulatory standards. The FDA appears to favor indication-focused benefit-risk assessments with biomarker integration based on robust, pre-specified subgroup analyses rather than requiring uniform efficacy across all populations.

**Figure 5:**
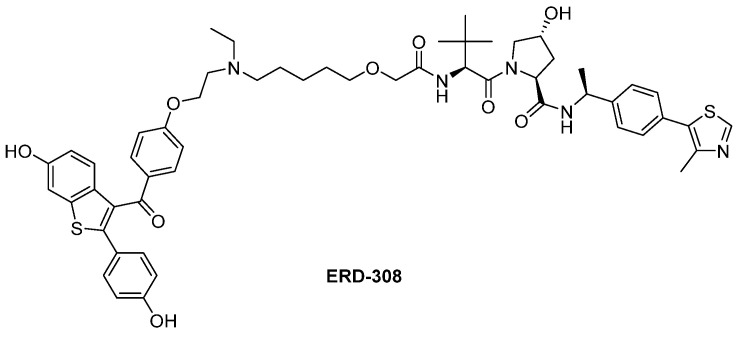
Structure of the investigational ER-targeted PROTAC ERD-308.

Several PROTACs have reached the clinical phase with different molecular targets, e.g., ARV-102, targeting leucine-rich repeat kinase 2, or gridegalutamide targeting the androgen receptor. However, other ER-targeted PROTACs besides vepdegestrant are quite few and have not progressed very far in clinical development. For example, AC682 demonstrated good ER degradation in preclinical studies with subnanomolar DC_50_ value and showed activity against clinically relevant ESR1 mutations. Its phase I study was, however, terminated, and until now no results have been made available. AC699 appears to be more promising, as it showed a 45% CBR and a 7.4-month progression-free survival in phase II results in ESR1 mutant patients, which are comparable or superior to those of vepdegestrant at the same stage of development. ERD-308 utilizes the cereblon/cullin 4A and VHL/cullin 2 neddylation degradation systems, and its ER-binding part is a raloxifene analog (Figure 5). It has a DC_50_ of 1.7 nM in MCF-7 cell line and induced more complete ER degradation than fulvestrant. Unfortunately, no clinical results are available about ERD-308. ERD-3111 and ERD-4001 showed excellent oral bioavailability and sub-nanomolar DC_50_ values in preclinical studies. They induced tumor regression in an in vivo model more effectively than vepdegestrant. HP568 is still in preclinical development and has shown favorable PK profile and strong synergism with CDK4/6 inhibitors [56,57,58,59,60].

Although more than 40 PROTAC degraders have entered human trials in oncology and other indications, the majority are still in phase 1–2, and vepdegestrant is the first to deliver positive phase 3 results and achieve regulatory approval. In contrast to vepdegestrant, which progressed through phase 3 with positive efficacy and a manageable safety profile in a biomarker-defined population, most other PROTACs have either remained in early phase evaluation or have been quietly discontinued, often without detailed public disclosure of failure modes. Available analyses suggest that suboptimal pharmacokinetics, off-target degradation, and inherent permeability and size constraints of the modality are frequent contributors to setbacks in preclinical and clinical setting in this class, rather than a single, well characterized ‘PROTAC specific’ failure pattern.

While ER-degrading PROTACs represent a new area and the drug candidates are in early stages of development, the older alternatives, SERDs, are more advanced in this aspect. For example, besides the already approved SERD drugs, camizestrant is under decision in the US and has received a positive recommendation by the EMA, while in the case of giredestrant, FDA decision is expected before the end of this year. The potential success of vepdegestrant may bring ER-targeting PROTACs to the forefront. However, it is possible that SERDs, with similar efficacy but simpler structures, will oust PROTACs from this indication. Maybe PROTACs are more promising in areas where there are no similar, protein degrader competition. Considering other indications, the main challenge of the development of oral PROTACs is the inherently complex structure, and the difficult pharmacokinetics which requires careful design and delicate balancing between the physicochemical properties of the molecule. A potential solution for this problem can be the use of click-formed proteolysis targeting chimeras (CLIPTACs), which unlike classic PROTACs are formed intracellularly via biorthogonal click reaction from smaller molecules [61].

Regarding the prospects for vepdegestrant, although the initial results are positive, several questions remain that will need to be answered over time, using results from greater studies with higher number of patients and longer follow-up periods. The latter should also clarify long-term tolerability, and the potential occurrence of drug resistance. Current studies also highlight the possibility of potential drug–drug interactions (DDIs). Although the studied DDIs did not raise serious safety concerns, careful monitoring may be necessary during vepdegestrant treatment to avoid and manage potential future interactions.

Still there are a few ongoing clinical trials with vepdegestrant. In the TACTIVE-U program, several 1b/2 studies have been started to investigate the efficacy of vepdegestrant in combination with kinase inhibitors (abemaciclib, ribociclib and samuraciclib). In the NCT06206837 1b/2 trial, the combination of vepdegestrant and atirmociclib (PF-07220060) is investigated. The VERITAC-3 study compares vepdegestrant + palbiciclib vs. letrozole + palbociclibe, but unlike the VERITAC-2, it investigates first-line settings (not pretreated patients). With approx. 1130 patients involved, VERITAC-3 could be the largest study of vepdegestrant [17].

Molecular analysis revealed that vepdegestrant has the potential to bind to rhotekin 2 (RTKN2), which is a protein, overexpressed in some gemcitabine-resistant cholangiocarcinoma [62]. Although this is only a computational evidence, future studies in this area may lead to the discovery of another potential indication of vepdegestrant. This could be another possible route to evolve into a first-line treatment, if VERITAC-3 shows satisfying results.

## Figures and Tables

**Table 1:** Comparison of different ER-targeting approaches.

	SERM	SERD	PROTAC
Complexity of the molecule	low	low	higher
Main mechanism of action	receptor antagonism/ agonism	decreasing receptor level	decreasing receptor level
Can act as antagonist?	yes	yes	yes
Leads to target degradation?	no	yes	yes
Type of mechanism	occupancy-driven	occupancy-driven	event-driven
Quantity required for degradation	-	stoichiometric	catalytic
Efficiency of degradation	-	lower	higher

**Table 2:** Molecular properties and Lipinski’s rule of five parameters of vepdegestrant.

Parameter	Lipinski Criterion	Vepdegestrant
Formula	-	C_45_H_49_N_5_O_4_
Mol. weight	<500	723.9180 g/mol
Number of HBDs	<5	2
Number of HBAs	<10	7
LogP	<5	6.7801
Polar surface area	<180	96 Å^2^
pKa	-	8.159

**Table 3:** Clinical efficacy of vepdegestrant and two approved modern, oral SERDs.

	Vepdegestrant	Elacestrant	Imlunestrant
Phase III trial	VERITAC-2	EMERALD	EMBER-3
Median follow-up	7.4 months	15.1 months	16.7 months
Median age of patients in the treated group	60.0 (26–87)	63 (24–89)	61 (28–87)
Number of all patients (treated vs. control)	313 vs. 311	239 vs. 238	331 vs. 330
Number of ESR1 mutant patients (treated vs. control)	136 vs. 134	115 vs. 113	138 vs. 118
Control group	fulvestrant	fulvestrant or aromatase inhibitor	fulvestrant or exemestane
PFS in overall patient population (treated vs. control) ^1^	3.8 vs. 3.6	2.8 vs. 1.9	5.6 vs. 5.5
PFS in ESR1 mutant subgroup (treated vs. control) ^1^	5.0 vs. 2.1	3.8 vs. 1.9	5.5 vs. 3.8

^1^ In months.

**Table 4:** Summarizing the most common adverse events of vepdegestrant and SERDs.

Adverse Effect % (All/Grade ≥ 3)	Vepdegestrant ^1^	Fulvestrant ^1,2^	Elacestrant ^2^	Imlunestrant ^3^
Any	86.9/23.4	81.4/17.6 ^1^ or 89.4/23.6 ^2^	92.0/28.7	82.6/17.1
Fatigue	26.6/1.0	15.6/1.3 ^1^ or 21.7/0.6 ^2^	19.0/0.8	22.6/0.3
Nausea	13.5/0	8.8/0.7 ^1^ or 16.1/0 ^2^	35.0/2.5	17.1/0.3
Anemia	12.2/1.6	7.8/3.3 ^1^	-	10.1/2.1
Neutropenia	11.5/1.9	4.6/1.0 ^1^	-	5.2/2.1
Back pain	10.9/0.6	6.5/0.3 ^1^ or 9.9/0.6 ^2^	13.9/2.5	10.7/0.6
Arthralgia	10.6/1.0	10.7/0 ^1^ or 17.4/0 ^2^	14.3/0.8	14.1/0.6
Decreased appetite	10.6/0.3	5.2/0 ^1^ or 7.5/0 ^2^	14.8/0.8	8.0/0.3
Increased alanine aminotransferase	14.4/0.6	9.8/0.7 ^1^ or 10.6/0 ^2^	9.3/2.1	10.4/0.3
Increased aspartate aminotransferase	14.4/1.3	10.4/2.6 ^1^ or 12.4/1.2 ^2^	13.1/1.7	12.5/0.9

^1^ Taken from the VERITAC-2 study, ^2^ Taken from the EMERALD study, ^3^ Taken from the EMBER-3 study.

## Data Availability

Data are contained within the article.

## Abbreviations

The following abbreviations are used in this manuscript:

AE	Adverse effect
AUC	Area under curve
BC	Breast cancer
BCRP	Breast cancer resistance protein
CBR	Clinical benefit rate
CDK 4/6	Cyclin-dependent kinase 4 and 6
CL	Clearance
Cmax	Maximal concentration
CRBN	Cereblon E3 ligase
CYP3A	Cytochrome P450 3A
DDI	Drug–drug interaction
DNA	Deoxyribonicleic acid
ER	Estrogen receptor
ER+	Estrogen receptor positive
ESR1	Estrogen receptor 1 gene
FDA	Food and Drug Administration
GI	Growth inhibition
HER2−	Human epidermal growth factor receptor 2 negative
HPLC	High performance liquid chromatography
HR+	Hormone receptor positive
HRMS	High-resolution mass spectrometry
i.d.	Intraduodenal
i.v.	Intravenous
MS	Mass spectrometry
mTOR	Mammalian target of rapamycin
NDA	New drug application
Peff	Effective permeability
P-gp	P-glycoprotein
PGR	Progesterone receptor
PI3K	Phosphoinositide 3-kinase
PK	Pharmacokinetics
PROTAC	Proteolysis targeting chimera
QD	Quaque die
Rac	Accumulation ratio
RTKN2	Rhotekin 2
SERD	Selective estrogen receptor degrader
SERM	Selective estrogen receptor modulator
t1/2	Half-life
t1/2eff	Effective elimination half-life
TEAE	Treatment-emergent adverse effect
TGI	Tumor growth inhibition
tmax	Time needed to reach maximal concentration
TPD	Targeted protein degradation
TRA	Total radioactivity
TRAE	Treatment-related adverse effect
WBC	White blood cell
wt	Wild type
